# A New Method for Ground-Based Optical Polarization Observation of the Moon

**DOI:** 10.3390/s24082580

**Published:** 2024-04-18

**Authors:** Weinan Wang, Jinsong Ping, Wenzhao Zhang, Mingyuan Wang, Hanlin Ye, Xingwei Han, Songfeng Kou

**Affiliations:** 1 Changchun Observatory, National Astronomical Observatories, Chinese Academy of Sciences, Changchun 130117, China; hanxw@cho.ac.cn; 2 University of Chinese Academy of Sciences, Beijing 101408, China; 3 National Astronomical Observatories, Chinese Academy of Sciences, Beijing 100012, China; wangmy@nao.cas.cn; 4 Astronomy Department, Beijing Normal University, Beijing 100875, China; wenzhaozhang@bnu.edu.cn; 5 International Center for Climate and Environment Sciences, Institute of Atmospheric Physics, Chinese Academy of Sciences, Beijing 100029, China; yehl@radi.ac.cn; 6 Nanjing Institute of Astronomical Optics and Technology, Chinese Academy of Sciences, Nanjing 210042, China; sfkou@niaot.ac.cn

**Keywords:** polarization imaging, polarimeter, white light, moon

## Abstract

As a natural satellite of the Earth, the moon is a prime target for planetary remote sensing exploration. However, lunar polarization studies are not popular in the planetary science community. Polarimetry of the lunar surface had not been carried out from a spacecraft until the Korean lunar exploration program was initiated. In previous polarization observations of the moon, images of different polarization states were obtained by a rotating linear polarizer. This method is not well suited for future polarization observations from space-based spacecraft. To this end, we present a new kind of polarized observation of the moon using a division of a focal-plane polarization camera and propose a pipeline on the processing method of the polarization observation of the moon. We obtain a map of the degree of white-light polarization on the nearside of the moon through polarization observation, data processing, and correction. The observation and data processing methods presented in this study have the potential to serve as a reference for analyzing polarization observation data from future orbiting spacecraft. These are expected to lead to new discoveries in the fields of astronomy and planetary science.

## 1. Introduction

Polarization, one of the intrinsic properties of light, can be used to describe the direction of vibration of light. Any target in the process of reflection, scattering, or electromagnetic radiation will produce polarization characteristics determined by its own nature.

In remote sensing applications, polarization imaging can enhance the discrimination of different surface types and improve the classification of land cover. In materials science, it can aid in the characterization of surface roughness and the detection of defects [1,2]. In biomedical imaging, it can assist in the visualization of tissue structures and the diagnosis of pathological conditions [3,4]. These diverse applications highlight the versatility and utility of polarization information across various disciplines. As technology continues to advance, the integration of polarization sensing capabilities into different imaging systems opens up new opportunities for research and innovation, further expanding the range of applications where polarization plays a crucial role.

The natural light emitted from the sun can be considered as unpolarized within $10^{-6}$th order of sensitivity [5], and when the light is reflected by the surface of a solid planet, a certain degree of polarization occurs. The moon is a popular object for space exploration, and its observation through ground-based telescopes remains attractive. Ground measurement methods are often used in space experiments. The optical properties of the moon have four basic characteristics: intensity, phase, albedo, and polarization of light. There is a long history of observations of lunar polarization. Lyot [6] carried out polarimetric observations of the moon in the 1920s. Kohan [7] studied the polarization characteristics of lunar surface strata and numerous Earth rocks based on Stokes parameters. Hapke [8] compared the optical properties of the lunar surface with those of rocks and meteorite powders. The Kharkov Observatory began a series of studies to analyze the polarimetric anomalies in the Umov effect [9,10]. Dollfus [11] followed with individual polarimetric measurements of several regions of the moon by means of video polarimetry. Korokhin et al. [12] carried out polarimetric observations of the Western hemisphere of the moon using the bands 4610 Å and 6693 Å and characterized the lunar surface using polarimetric color-ratio images. Shkuratov et al. [13], on the other hand, carried out polarimetric observations of the Western hemisphere of the moon using the 4200 Å and 6500 Å bands and analyzed the characteristics of the lunar surface through polarimetric colour-ratio images. The colour-ratio images obtained through multispectral polarimetry were utilized to study the granular structure of the lunar regolith on the moon. The polarimetric anomaly parameter ${\left(P_{max}\right)}^{a}A$ was utilized to characterize the lunar regolith [10,11]. It was discovered that the colour-ratio images were susceptible to the thickness of the lunar regolith grains on the surface. These studies concluded that the polarization data on the lunar surface contain information on the median particle size of the lunar regolith and the internal opacity of lunar regolith layer particles. Based on these findings, Minsup Jeong et al. [14] carried out visible multicolour polarimetric observations to obtain parameters such as the polarization degree (Pmax) and the albedo (A) of the nearside lunar surface. They established a median particle size map of the lunar frontal surface in order to investigate the lunar soil’s grain size distribution characteristics [15]. On the other hand, Andrew Fearnside et al. [16] proposed a method for the remote measurement of the refractive index of the lunar regolith via lunar polarization anomaly parameters, although it may still be flawed [17].

In the above polarimetric observations, multiple images of the moon in different polarization directions needed to be taken at different times [11,14], but this potentially increased the alignment error. Due to the technological advances in fine micro-nano-processing techniques, high-resolution polarization imaging has become feasible [18,19,20,21].

A division of the focal-plane polarization (DoFP) sensor uses a micro-optical array of polarization elements to make different polarization measurements at each pixel on the focal-plane array. The DoFP sensor makes it possible to acquire images in four linearly polarized intensities simultaneously. This is advantageous for observing dynamic and large-scale objects and for deployment on spacecraft. The drawback is that the DoFP sensor cannot acquire circular polarization, and the circular polarization of the moon (the forth Stokes parameter) is too small to be used as a tool for surface diagnostics [22]. Therefore, we propose a method to carry out ground-based remote sensing imaging of white-light polarization on the moon by using the DoFP camera. In this paper, we introduce the observing equipment and the process of carrying out polarimetric observations, and the data processing method is presented. Finally, we obtain the degree of polarization map of the white light and present the situation of the lunar surface.

## 2. Materials and Methods

### 2.1. Principle of Division of Focal-Plane Polarization Sensor

**Figure 1:**
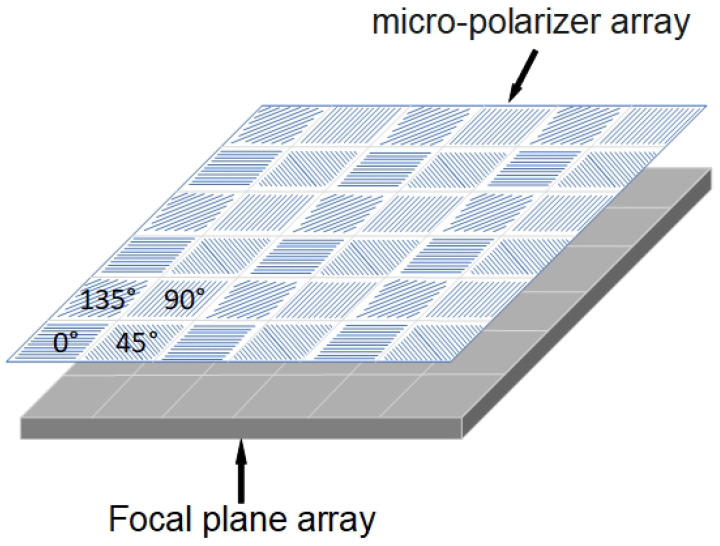
Schematic structure of a DoFP sensor.

DoFP is a pioneering technology that integrates a complementary metal oxide semiconductor (CMOS) image sensor with in-pixel capabilities. This innovative approach involves the incorporation of multiple-angle polarizers directly onto the chip during the semiconductor manufacturing process, ensuring precise alignment with each pixel. By incorporating polarizing elements within individual pixels, the DoFP sensor can capture polarization information along with conventional intensity data, enabling advanced imaging capabilities with an enhanced depth of field. Micropolarizer arrays with a four-directional polarizer (0°, 45°, 90°, and 135°) are obtained at the pixel level by inscribing different directional line grids on the photoreceptor chip. Each array corresponds individually to each pixel of the image sensor in Figure 1.

The four-directional polarizer corresponding to each neighbouring pixel forms a “super-pixel” to characterize the polarization information. The strategic placement of polarizers at the pixel level enhances the accuracy and efficiency of polarization imaging, allowing for a detailed analysis of polarized light across various angles. This unique configuration enables DoFP sensors to capture rich polarization information while maintaining high spatial resolution, making them well suited for applications requiring simultaneous intensity and polarization data acquisition. Compared to time-dependent polarization filter imaging, a DoFP sensor avoids the fringing effect caused by the apparent libration of the moon and the variation in the moon’s phase angle due to the large interval between the observation of the four polarization direction images. It allows all polarization parameters to be measured simultaneously in a single image, which facilitates continuous polarization observations of celestial bodies.

**Figure 2:**
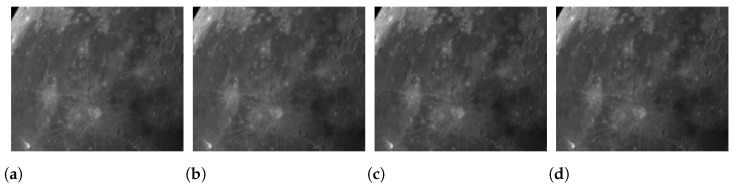
Four-directional polarization information captured in one shot: (**a**) 0-degree polarization direction sub-image; (**b**) 45-degree polarization direction sub-image; (**c**) 90-degree polarization direction sub-image; (**d**) 135-degree polarization direction sub-image.

The polarization light can be represented by Stokes parameters, and its polarization degree *P* can be expressed as

(1) $$P=\frac{\sqrt{Q^{2}+U^{2}}}{I}$$

where *U*, *I* are Stokes parameters, respectively. They are

(2) $$I=\frac{I_{0^{\circ }}+I_{45^{\circ }}+I_{90^{\circ }}+I_{135^{\circ }}}{2}$$

(3) $$Q=I_{0^{\circ }}-I_{90^{\circ }}$$

(4) $$U=I_{45^{\circ }}-I_{135^{\circ }}$$

where $I_{0^{\circ }}$, $I_{45^{\circ }}$, $I_{90^{\circ }}$, and $I_{135^{\circ }}$ represent the sub-images of light intensity corresponding to the polarization directions of 0°, 45°, 90°, and 135° of the polarizer, respectively, as shown in Figure 2.

### 2.2. Observation

In this study, we presented the first white-light polarization observations of the moon by using a Schmidt–Cassegrain telescope in Xinjiang, China, from August to October 2022. A motorized EQ5 equatorial mount allowed for the tracking of the relative lunar motion, with manual corrections applied by the arrow keys of its hand controller when observing different lunar surface regions.

**Figure 3:**
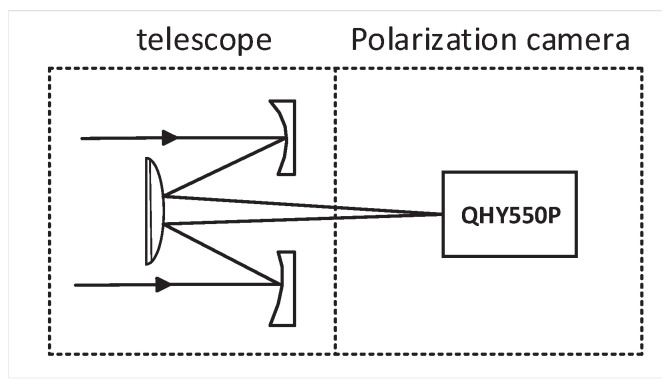
Optical system configuration of the experiment mounting.

We used a QHY550P DoFP camera. It has an analog-to-digital (A/D) sampling depth of 12 effective bits. The camera adopts a Sony IMX250 MZR (Tokyo, Japan) polarized chip and the full resolution stands at 2460 × 2070. The polarization image sensors has a multi-directional polarizer, which is formed on top of the photodiode of the image sensor chip (on-chip polarizer). The basic schematic of the optical system is shown in Figure 3.

We set the polarization camera gain between 1 and 50, which should not be changed for the observation night. The average count value of images is controlled to be between 40,000 and 55,000 to select the proper exposure time and the camera gain parameter. Different exposure times are employed for varying phases of the moon, with each frame of the image typically having an exposure time of approximately 20–30 ms. Specific parameters guiding our observations are meticulously detailed in Table 1.

### 2.3. Auxiliary Images

Before the start of polarization observations, it is necessary to have some auxiliary images for instrumental calibration. Astronomical auxiliary images include bias, dark-field and flat-field images. A bias is an image taken with no light falling on the CMOS sensor. Bias refers to the direct-current bias attached to the readout signal during a driven readout. It is an intrinsic reading of a CMOS sensor due to the bias voltage and inherent structure. A dark-field image is an image of the heat, the hot and cold pixels, random noise, and other camera characteristics, and is taken with a camera with the telescope cap on. Both flat-field and observation images need to be subtracted before they can be processed. Before each night’s observation, ten frames each of bias and dark-field are captured, contingent upon the camera parameters applied for lunar observation.

A flat-field image is used to correct for unevenness in the optical path caused by mirrors, optics, and in the pixel response of the CMOS sensor itself. Flat-field images are taken at twilight when the incident light in the sky is uniform and bright enough to reach the 1/2 full-well-capacity limit. These images are also used identically to the camera parameters.

### 2.4. Polarization Error Calibration

The DoFP camera features robust, well-aligned designs and possess inherent temporal synchronization. These attributes render the DoFP camera the optimal choice for real-time imaging applications. Also referred to as microgrid polarimeters, these instruments, with their snapshot nature and absence of moving components, mitigate challenges commonly associated with traditional polarimetry. Issues like vibration, spurious signals, beam wander, and the necessity for registration routines are effectively eliminated. However, the performance of DoFP polarimeters is frequently constrained by various error sources, including fixed pattern noise (FPN), photon response nonuniformity (PRNU), nonuniformity in micropolarizer extinction ratio, micropolarizer orientation misalignment, pixel cross-talk, and instantaneous field of view (IFOV) error [20].

To measure and correct the inherent polarization error in the telescope system, a calibration was implemented before conducting lunar polarization observations. During clear nights, specific polarization standard stars were chosen for calibration purposes, ensuring minimal disparity of the telescope system. The calibration procedure involved the following steps: First, we observe a polarization standard star and the nearby sky light background. Then, we observe a zero-polarization standard star and the nearby sky light background. Relative calibration [23,24] is performed by observing the polarization standard stars in the Heiles catalog [25] and the zero-bias standard stars taken from the Hubble Space Telescope atlas [26]. Specific standard stars guiding our observations are meticulously detailed in Table 2. These non-polarized standard stars are used to measure the instrumental polarization introduced by the telescope and optical instrument. These standard stars are generally imaged close to the array’s center and can be used to correct the measured Stokes parameters before our lunar observations.

The typical uncertainty of the obtained degree of polarization of white light is not larger than 0.5% for each frame by relative calibration.

## 3. Data Processing

**Figure 4:**
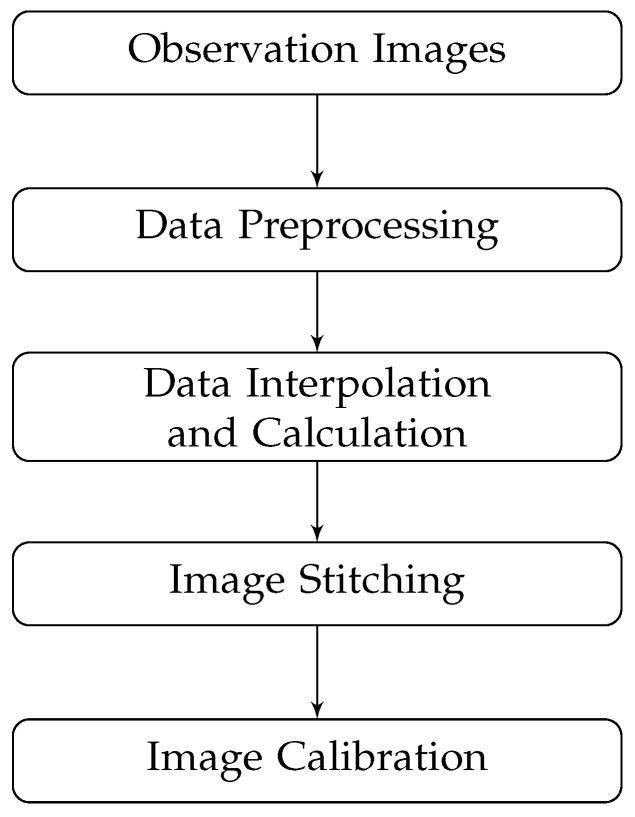
The flowchart of observation processing.

After each night’s observation, the raw data are processed in a structured routine as shown in Figure 4. The flowchart primarily contains several integral steps for data processing: data preprocessing, data interpolation and calculation, image stitching, and image correction. It is important to note that after acquiring four images of light intensity in different polarization directions, each of them was utilized separately.

### 3.1. Data Preprocessing

**Figure 5:**
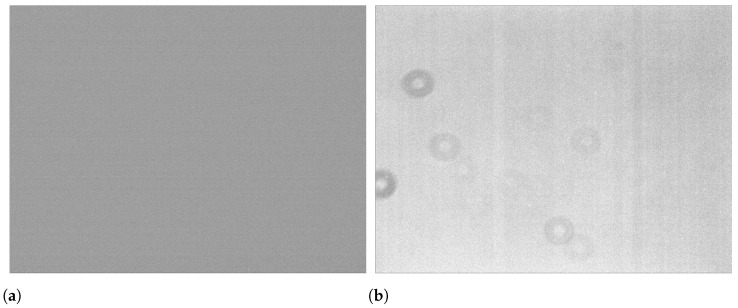
Auxiliary bias and flat images. (**a**) Combined bias: median image of 10 bias images. (**b**) Combined flat:median image of 10 flat-field images.

To mitigate the thermal noise in the polarization camera, as well as observing equipment systematic errors and cosmic ray effects, a data preprocessing pipeline was employed, as shown in Figure 5. The image data corresponding to all Stokes parameters undergo individual processing. This process includes the subtraction of bias and flat-field images, performed using Equation (5):

(5) $$Image^{\prime }=\frac{Image-Bias}{normalized\;(Flat-Bias)}$$

where $Image^{\prime }$ represents the corrected image, and Image represents an original observation image. The median of the ten bias and flat-field images are separately merged to obtain a single image as Bias and Flat.

### 3.2. Data Interpolation and Calculation

**Figure 6:**
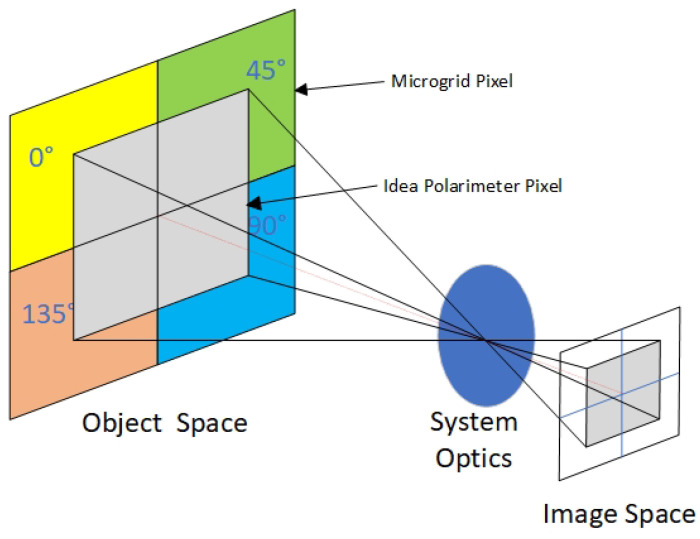
Schematic diagram of the instantaneous field of view error in the defocusing plane [27]. The ideal polarimeter pixel is capable of measuring the same area in space over the same interval in time for each polarizer orientation. IFOV error arises due to differences in the captured spatial areas or timing intervals captured by the ideal polarimeter pixel and the microgrid pixels.

Due to the limitation of each frame element in measuring only one of the three necessary polarization intensity measurements required for the Stokes vector at each position, inherent challenges arise. Consequently, this restriction leads to a compromise in imaging resolution and deviation in the instantaneous field of view (IFOV) due to potential misalignment among pixels, as illustrated in Figure 6. After the correction of four directional polarization light intensity sub-images by image preprocessing, bicubic spline interpolation is used to reduce the negative effects of diminished spatial resolution and the IFOV error. The bicubic spline interpolation method achieves a continuous curve and surface representation, resulting in a smooth image while preserving intricate detail and minimizing jaggy effects. This method is superior to bilinear and nearest-neighbor interpolation methods [19].

Before this interpolation process, an extraction of pixel values corresponding to the micropolarizer arrays with identical polarization directions is carried out. This extraction results in the creation of a quart-sized sub-image, essentially representing the light intensity image for each distinct polarization direction in Figure 2. Subsequently, bicubic spline interpolation is applied to these four individually corrected sub-images, namely $I_{0^{\circ }}$, $I_{45^{\circ }}$, $I_{90^{\circ }}$, and $I_{135^{\circ }}$, where each one is associated with a specific polarization direction. The basic steps involved in the implementation are as follows. The original image is (x, y) and the image to be interpolated is $\left(x^{\prime },y^{\prime }\right)$. Then, we obtain the pixel values of point $\left(x^{\prime },y^{\prime }\right)$ by interpolating the pixel values of the 4 × 4 integer points around the point (x,y) using Equation (6).

(6) $$I\left(x^{\prime },y^{\prime }\right)=\sum_{i=0}^{3}\sum_{j=0}^{3}I\left(i,j\right)\times W(i,j)$$

where *W* is the weight function, defined as W(i,j):

(7) $$W\left(i,j\right)=w\left(d_{i}\right)\times w(d_{j})$$

w(d) is the bicubic spline interpolation base function, defined as

(8) $$w\left(d\right)=\left\{\begin{array}{cc} \frac{2}{3}-\left(1-\frac{\left|d\right|}{2}\right)\times {\left|d\right|}^{2} & ,|d|\leq 1\\ \frac{1}{6}{\left(2-|d|\right)}^{3} & ,1<|d|\leq 2\\ 0 & ,\mathrm{else} \end{array}\right.$$

(9) $$\left\{\begin{array}{c} d_{i}=p\left(i,j\right).x-x^{\prime }\\ d_{j}=p\left(i,j\right).y-y^{\prime } \end{array}\right.$$

where p(i,j).x and p(i,j).y are the x-coordinate and y-coordinate of the point p(x,y), respectively.

**Figure 7:**
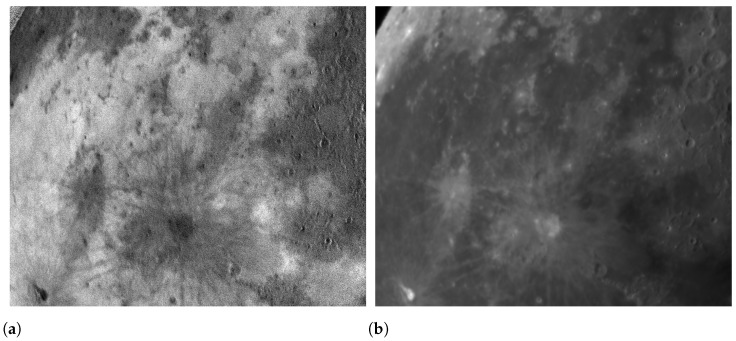
DOP image and light intensity image are calculated by the Stokes formula. (**a**) DOP image. (**b**) Light intensity image.

Upon completing the interpolation procedure for these sub-images, the resulting interpolated sub-images maintain the same size as the original image. This approach ensures the alignment of the interpolated sub-images with the original image, effectively capturing and preserving the intricate polarization details while maintaining consistency in the overall image dimensions [19,27]. Then we apply Equations (1) and (2) to the images for each directional polarization to obtain the corresponding degree of polarization (DOP) image and intensity image in Figure 7, respectively.

### 3.3. Image Stitching and Geometric Calibration

The FOV of the telescope system is $12.43^{\prime }\times 10.37^{\prime }$, as described in Table 1, much smaller than the apparent diameter of the moon. It is not enough to cover the entire lunar surface. The observed images of the whole region of the lunar surface need to be mosaiced and geometrically calibrated for subsequent analysis.

**Figure 8:**
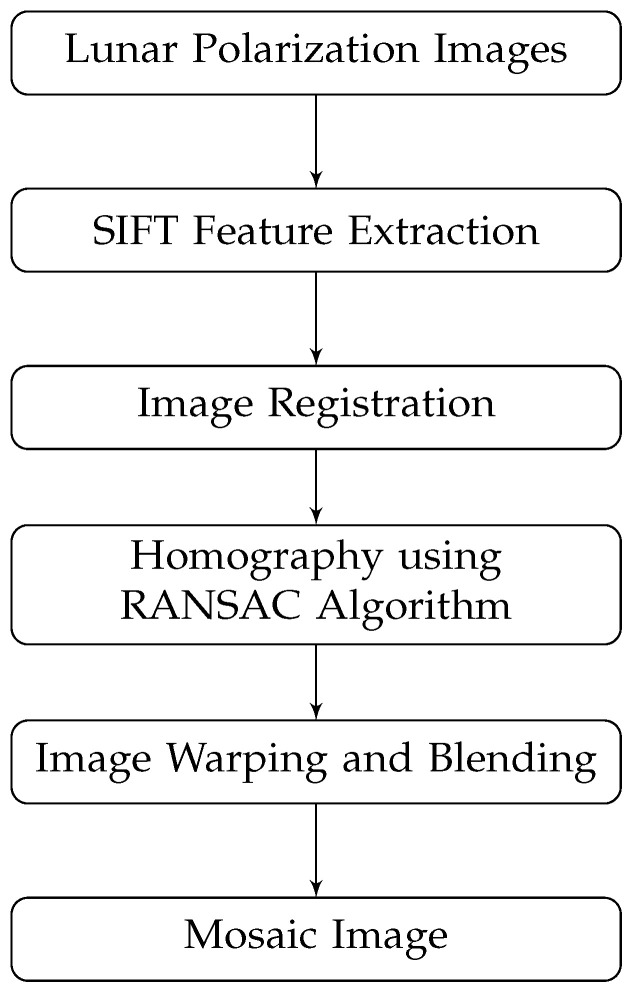
The flowchart of image mosaicing [32,33].

The SIFT and RANSAC algorithms are used to obtain the mosaic of lunar polarization images [28,29]. The SIFT (Scale-Invariant Feature Transform) is an algorithm for detecting and describing local features in the image, which is scale-invariant and robust to rotation, scaling, luminance-changing, and viewing angle-changing [30,31]. The flow of these processes is shown in Figure 8. The initial step is to create a SIFT object to detect feature points and compute their descriptors. Then, establish a FLANN matcher to match these feature descriptors. After that, extract the accurate matching point pairs using the RANSAC algorithm. Based on these matching point pairs, compute the respective transformation matrix and mosaic images together.

Then, the geometric calibration of the lunar image can be achieved based on the mosaic image of the moon and the inner and outer orientation elements of the telescope system. The inner orientation elements of the telescope system include the camera’s principal point, focal length, and distortion. images can be converted from the central projection to the orthographic projection to reduce the aberrations at the edges. The external orientation elements such as telescope position and azimuth angle can be used to transform the image point from the image coordinate system to the real-world coordinate system. According to the internal and external orientation elements, the image point coordinates are converted to the lunar solid coordinate system. The coordinates of the telescope in the lunar solid coordinate system are found, and it is known that the three points of the lunar surface point, the image point, and the telescope are co-located, so that the localization model can be constructed directly into Equation (10):

(10) $$\left[\begin{array}{c} X\\ Y\\ Z \end{array}\right]=\left[\begin{array}{c} Xs\\ Ys\\ Zs \end{array}\right]+m\left(R_{MCI}^{MCMF}R_{J2000}^{MCI}R_{Body}^{J2000}\right)R_{Camera}^{Body}\left[\begin{array}{c} x\\ y\\ f \end{array}\right]$$

where ${\left[XYZ\right]}^{T}$ denotes the coordinates of the lunar surface point under the lunar solid coordinate system at the center of the moon. ${\left[XsYsZs\right]}^{T}$ represents the coordinates of the telescope under the lunar solid coordinate system at the imaging moment. ${\left[xyf\right]}^{T}$ is the coordinates of the image point under the telescope coordinate system. $R_{Camera}^{Body}$ is the transformation matrix from the telescope coordinate system to the body coordinate system. $R_{Body}^{J2000}$ is the rotation matrix from the body coordinate system to the J2000 coordinate system. $R_{J2000}^{MCI}$ is the rotation matrix from the J2000 coordinate system to the moon-centered inertial (MCI) frame, and $R_{MCI}^{MCMF}$ is the rotation matrix from the moon-centered inertial (MCI) frame to the moon-centered moon-fixed (MCMF) frame. The orientation of the MCMF and MCI frame is realized by DE430 adoptions [34].

**Figure 9:**
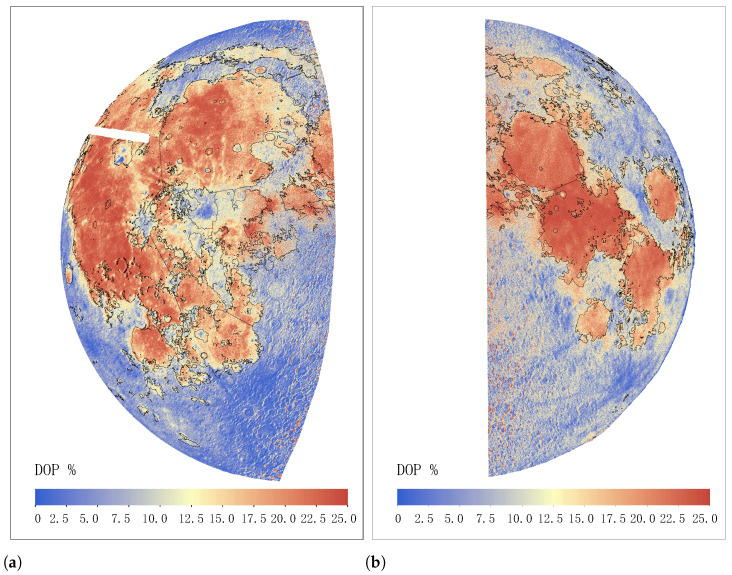
The degree of polarization map from the Eastern (**a**) and Western (**b**) hemispheres using our observation data. The digitization of lunar mare boundaries is represented by the black solid line [35].

By following the above steps, we mosaiced and calibrated these frames of the same night into one image and obtained the DOP map, as shown in Figure 9. Figure 9a,b show observations during the last and first quarter moons, respectively.

When imaging a target, the target pixel resolution (GSD) is

(11) $$GSD=\frac{aL}{F}$$

where *a* is the pixel size of the image detector, *F* is the focal length of the telescope, and *L* is the vertical distance to the target (Earth–moon distance). For our observation equipment in Table 1, the theoretical spatial resolution of each pixel is 1.324 × 103 m. Due to the influence of atmospheric turbulence and system error, the mean spatial resolution of the white light DOP map is 1.58 km/pixel.

## 4. Results and Conclusions

From the DOP map, we can observe differences in the polarization degree of white light in different regions. The polarization degree of white light in the Maria region of the moon is significantly higher than that in the lunar highlands, which may be related to the geological composition and surface features of the region. In the oceanic troposphere region, the value of white light polarization reaches a peak of nearly 20%, indicating that the region contains more components of polarized light. In addition, we found that near the boundary line of the moon, especially the position of the lunar mare, there is also a high degree of white light polarization. This high polarization distribution may be related to the formation of landforms in the region, as there are usually some geological activities near the lunar boundary. According to the analysis of the DOP map, we also observed that the Western hemisphere of the moon has a stronger degree of white light polarization than the Eastern hemisphere. This can be explained by the relationship between the observation period and the lunar phase angle, leading to this difference. When the observation period approaches the last quarter of the lunar phase angle, we can observe an increase in white light polarization in the Western hemisphere, while in the Eastern hemisphere, due to the observation period approaching the weakened convex angle, white light polarization is weaker. Meanwhile, we also found that DOP maps can be used to identify terrain features such as volcanic craters and impact debris on the moon. These features may have different polarization characteristics and are worth further investigation. By analyzing the DOP map, we can further understand the surface composition and formation history of the moon.

In this paper, a new method for observing the moon using a DoFP camera is proposed. This paper details the data processing pipeline for ground-based polarimetric observation of the moon. The performance of lunar observations with the DoFP camera is a first attempt, and this method has unique advantages over conventional polarimetry: the alignment process can be affected by an “edge effect” induced by lunar libration and shifts in phase angle, which are influenced by the lunar surface’s topographical features. This new polarization observation method avoids the edge effect and not only improves the accuracy and efficiency of lunar surface observations, but also creates new opportunities for future polarimetric observations of dynamic objects and large regions on spacecraft.

## Figures and Tables

**Table 1:** Techniques parameters of observation equipment.

Parameter	Value
Telescope diameter	0.235 m
Telescope focal length	2.35 m
Field of view (FOV)	$12.43^{\prime }\times 10.37^{\prime }$
Pixel scale	0.61 arcsec
Frame size	2460×2070
Wavelength	400 nm to 800 nm

**Table 2:** The degree of polarization for observations.

Name	RA/DEC (J2000)	P (%)
HD12021	01 h 57 m 56.14 s/−02°05′57.727″	0.04
HIP746	00 h 09 m 11.524 s/+59°08′54.866″	0.01
HIP112029	22 h 41 m 27.845 s/+10°49′52.648″	0.03
HD7927	01 h 20 m 04.914 s/+58°13′53.746″	3.31
HD204827	21 h 29 m 36.030 s/+58°50′43.148″	5.43
HD183143	19 h 28 m 28.936 s/+18°20′32.653″	4.95

## Data Availability

The data presented in this study are available on request from the corresponding author.
